# Human DNA contamination of postmortem examination facilities: Impact of COVID‐19 cleaning procedure

**DOI:** 10.1111/1556-4029.15096

**Published:** 2022-07-18

**Authors:** Carla Bini, Arianna Giorgetti, Elena Giovannini, Guido Pelletti, Paolo Fais, Susi Pelotti

**Affiliations:** ^1^ Department of Medical and Surgical Sciences, Unit of Legal Medicine University of Bologna Bologna Italy

**Keywords:** autopsy, COVID‐19, DNA contamination, forensic genetics, forensic pathology, q‐PCR

## Abstract

The DNA contamination of evidentiary trace samples, included those collected in the autopsy room, has significant detrimental consequences for forensic genetics investigation. After the COVID‐19 pandemic, methods to prevent environmental contamination in the autopsy room have been developed and intensified. This study aimed to evaluate the level of human DNA contamination of a postmortem examination facility before and after the introduction of COVID‐19‐related disinfection and cleaning procedures. Ninety‐one swabs were collected from the surfaces and the dissecting instruments, analyzed by real‐time quantitative PCR (q‐PCR) and typed for 21 autosomal STRs. Sixty‐seven out of 91 samples resulted in quantifiable human DNA, ranging from 1 pg/μl to 12.4 ng/μl, including all the samples collected before the implementation of COVID‐19 cleaning procedures (*n* = 38) and 29 out of 53 (54.7%) samples taken afterward. All samples containing human DNA were amplified, resulting in mixed (83.6%), single (13.4%), and incomplete (3%) profiles. A statistically significant decrease in DNA contamination was found for dissecting instruments after treatment with chlorhexidine and autoclave (*p* < 0.05). Environmental decontamination strategies adopted during COVID‐19 pandemic only partially solved the long‐standing issue of DNA contamination of postmortem examination facilities. The pandemic represents an opportunity to further stress the need for standardized evidence‐based protocols targeted to overcome the problem of DNA contamination in the autopsy room.

**Highlights**

- Samples collected in the autopsy room showed human DNA contamination.
- Environmental decontamination from viruses does not affect human DNA.
- Chlorhexidine and autoclave resulted in DNA removal from dissection instruments.
- Protocols for prevention of DNA contamination in the autopsy room are needed.

## 1 INTRODUCTION

It is well known that forensic science DNA profiling has a central role in the criminal justice community helping conviction of the guilty and exoneration of the innocent even in capital offenses. However, the DNA profiling from biological material detected at a crime scene should always be interpreted with caution, considering that small amounts of “innocent DNA” could be found. The increased sensitivity of PCR‐based methodologies has enabled genetic profiles to be obtained from degraded samples or from trace samples left by talking, sneezing, skin cells shedding, and DNA left on the surfaces by touch—so called “touch DNA” [1, 2]. However, this has also exacerbated the issues of DNA persistence, background level and contamination [3, 4]. Given that DNA transfer might occur as a consequence of criminal or noncrime‐related activities, such as in contamination events, the main issue in forensic is represented by the mechanisms or actions that led to the deposition of the biological material concerned [5, 6]. Recently, the DNA commission of the International Society for Forensic Genetics (ISFG) published recommendations for forensic geneticists to evaluate DNA and biological results, whose value is impacted by phenomena such as secondary (or tertiary) transfer, contamination or “fortuitous” presence of DNA in the environment [7, 8].

The risk of contamination by exogenous DNA has been highlighted for samples collected from clothes or body surfaces of the victim in the mortuary, even if autopsy surfaces and instruments might appear falsely “clean” [9, 10, 11]. The DNA transfer at postmortem facilities does not represent a new matter, but the “mobility” of DNA is still an issue in court and a subject of research, as underlined by recent publications showing that DNA might be distributed even in the context of cleaning scenarios [12].

The COVID‐19 pandemic outbreak has led to significant changes in the autopsy practices: personal protective equipment, hygiene precautions, hospital disinfection, and sterilization methods, including those related to autopsy room, have been recently updated and intensified, all of them devoted to the prevention of the infectious risk [13, 14, 15, 16, 17].

Given the long‐standing issue of exogenous background DNA contamination and the recent attention devoted to the environmental cleaning from SARS‐CoV‐2, this study aimed to evaluate the level of human DNA contamination of a postmortem examination facility before and after the introduction of COVID‐19‐related disinfection and cleaning procedures, in order to assess their impact on the genetic typing of forensic evidentiary traces.

## 2 MATERIALS AND METHODS

### 2.1 Study design

The study was performed at the local postmortem examination facility of the University of Bologna, which is in use by several forensic pathologists, technicians, and occasionally by clinical pathologists.

Samples were collected from surfaces and dissecting instruments across 9 unannounced visits (V), which took place without any warning to pathologists and/or to cleaning services during two different time periods. Visits took place before the outbreak of COVID‐19 pandemic (V1–V3) and after the implementation of a COVID‐19 cleaning and decontamination plan (V4–V9) and were scheduled as follows:

- V1, V4, and V7 on a random day, assumed to be representative of the daily forensic routine;
- V2, V5, and V8 were done immediately before a scheduled postmortem examination;
- V3, V6, and V9 took place after the scheduled postmortem examination, as soon as a complete cleaning and drying of room and instruments was achieved.

### 2.2 Cleaning and decontamination procedures

#### 2.2.1 Surfaces

Before the pandemic, all surfaces of the facilities were routinely cleaned with the following products:

- Sanet Zitrotan (purchased from Werner & Merz Professional Srl, Milan, Italy), acid sanitary maintenance cleaner (5%–10% diluted solution) containing ethoxylated alcohols, sulfates, and sodium salts;
- Antisapril Clorossidante disinfectant (purchased from Amuchina, Rome, Italy), containing active chlorine 2.7% (sodium hypochlorite), traces of hypochlorous acid, and sodium chloride 8%. The disinfectant is active for Gram + and for alcohol‐acid resistant bacteria, protozoa, fungi, spores, virus, including HIV and Hepatitis B.

Cleaning products were provided diluted to a final sodium hypochlorite concentration of 0.1% and wiped up with absorbent material.

After the implementation of a COVID‐19 cleaning and decontamination plan, the following product was added: Sanet Br75 (purchased from Werner & Mertz Professional Srl) containing orthophosphoric acid (20%–25%), alcohols C13‐15, benzenesulfonic acid, C10‐13 alkyl derivatives, sodium salts, <5% anionic surfactants, and nonionic surfactants. This was diluted to an orthophosphoric acid concentration of 0.2%.

#### 2.2.2 Dissection instruments

Before COVID‐19 outbreak, dissection instruments were cleaned within the postmortem examination facilities immediately after the autopsy, with abundant water and a detergent consisting of 5%–15% anionic surfactants, <5% amphoteric surfactants, hydantoin, linalool, methylchloroisothiazolinone, and methylisothiazolinone, until the removal of visible staining. After the implementation of a COVID‐19 cleaning and decontamination plan, instruments were cleaned by the hospital services using Chlorhexidine gluconate and finally sterilized by autoclave.

The post‐COVID cleaning procedures followed the recommendations of the WHO [13] and, at a national level, those of the Italian National Institute of Health, of the Scientific Society of Hospital Legal Medicine of the National Health System and the Italian Society of Anatomical Pathology and Cytology (SIAPEC) [15, 16, 17].

### 2.3 Sampling

**FIGURE 1:**
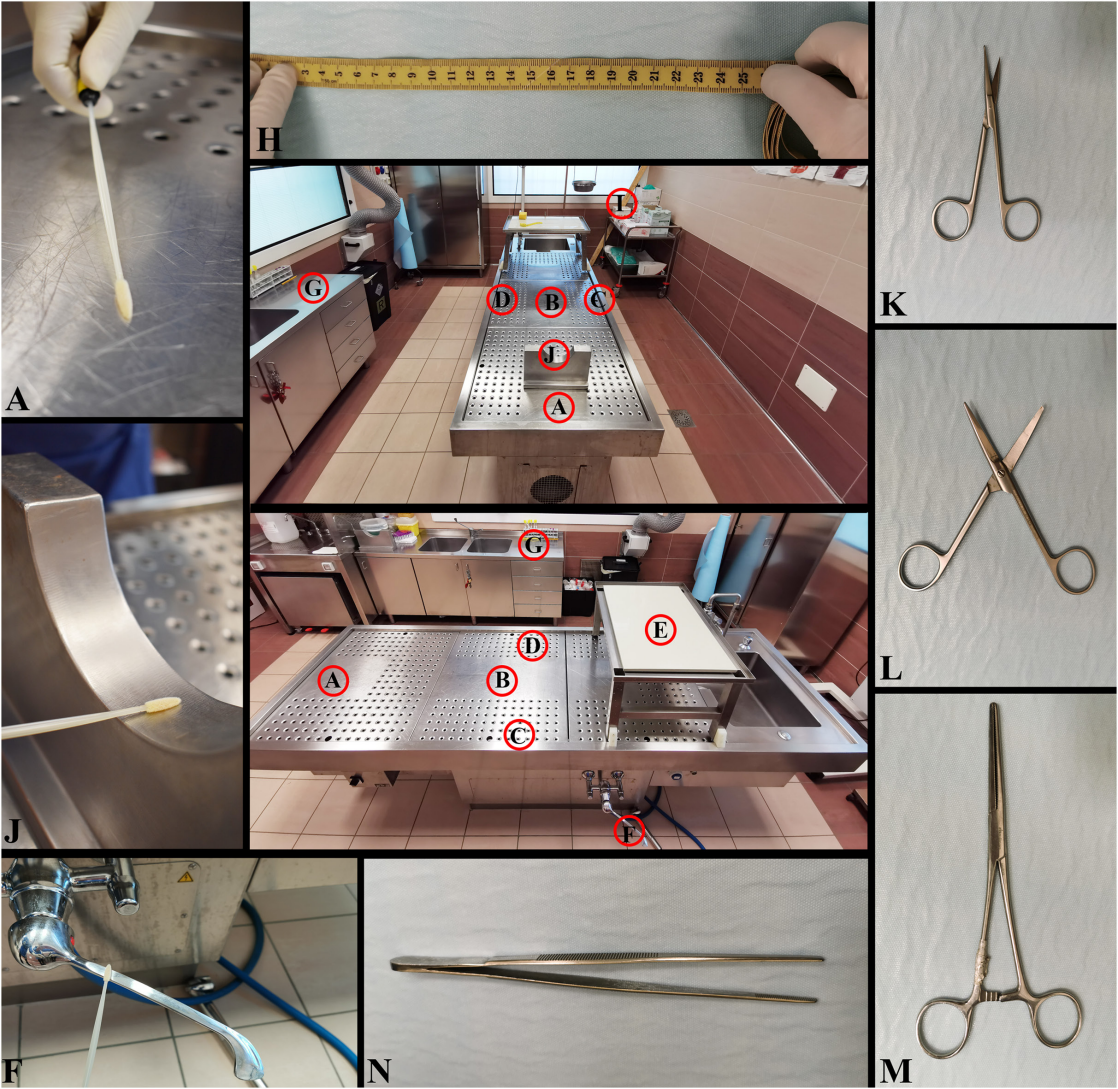
Surfaces and instruments sampled at the postmortem facility. A: autopsy table at the head level; B: autopsy table at the buttocks level; C: autopsy table at the right‐hand level; D: autopsy table at the left‐hand level; E: removable table; F: water tap; G: side table; H: measuring tape; I: glove box; J: head/neck support; K: coronary artery scissor; L: large‐size scissors; M: kocher; N: forceps.

Ten (*n* = 10) surfaces and four (*n* = 4) dissection instruments, which were assigned a letter from A to N, were swabbed for sampling. Details of the items, as well as the number of sampling per item, are shown in Figure 1 and Table 1.

**TABLE 1:** Site and number (n) of samples collected during nine unannounced visits (V) before (V1–V3) and after (V4–V9) the outbreak of the Covid‐19 pandemic

	Item	Site of sampling	V1–V3	V4–V9
*Surfaces*	*A*	Autopsy table at the head level	*n* = 3	*n* = 3
	*B*	Autopsy table at the buttocks level	*n* = 3	*n* = 3
	*C*	Autopsy table at the right‐hand level	*n* = 3	*n* = 3
	*D*	Autopsy table at the left‐hand level	*n* = 3	*n* = 3
	*E*	Removable table, used for the organs sectioning	*n* = 3	*n* = 3
	*F*	Water tap of the table	*n* = 3	*n* = 3
	*G*	Side table, above which dissecting instruments are usually placed during autopsies	*n* = 3	*n* = 3
	*H*	Measuring plastic tape, 130 cm long	*n* = 3	*n* = 3
	*I*	Glove box, medium size	*n* = 3	*n* = 3
	*J*	Head/neck support	*n* = 3	*n* = 3
*Dissection instruments*	*K*	Coronary artery scissors	*n* = 2	*n* = 5
	*L*	Medium‐size scissors	*n* = 2	*n* = 6
	*M*	Kocher	*n* = 2	*n* = 6
	*N*	Forceps	*n* = 2	*n* = 6
*Total*			*n = 38*	*n = 53*

Approximately 10 cm of surfaces and the whole cutting/contact area of dissection instruments were sampled using 4N6FLOQSwabs® Crime Scene. No macroscopical stains were visualized prior to samplings.

During V1, only surfaces samples A–J were available, since dissection instruments were not usually left in the local facility and were usually brought to the mortuary immediately before the autopsy.

On the basis of preliminary results, only dissection instruments were swabbed during V7–V9.

### 2.4 Genetic analyses

DNA extraction was performed using the QIAamp® DNA Investigator Kit (Qiagen) following the manufacturer's protocol for “isolation of total DNA from surface and buccal swabs” with a final ATE buffer elution volume of 25 μl. A negative control (extraction blank) was included in each extraction batch and analyzed alongside samples. All the DNA extracts were stored at −20°C until use.

The total amount of human DNA, human male DNA, as well as the quality of all the DNA extracts were assessed by real‐time quantitative PCR (q‐PCR) on the QuantStudio 5 Real‐Time PCR System (Applied Biosystems) using the PowerQuant® System (Promega) following the manufacturer's instructions.

PCR amplification was performed on samples showing the presence of human DNA >1 pg/μl using Veriti^™^ 96‐well Thermal Cycler (Thermo Fisher) and GlobalFiler^™^ IQC PCR Amplification Kit (Thermo Fisher) in a final reaction volume of 5 μl by adding up to 250 pg of DNA input. A positive DNA Control 007 (Thermo Fisher), as well as extraction blanks and a nontemplate control were added in each PCR batch.

PCR products were separated and detected by capillary electrophoresis on the SeqStudio^™^ Genetic Analyzer (Applied Biosystems) following the manufacturer instructions. GlobalFiler^™^ Allelic Ladder was included in each capillary electrophoresis run.

Data collection software was used to collect raw data and genemapper idx v 1.6 (Thermo Fisher) for allele calls and profile analysis, using an analytical threshold value of 100 RFU.

### 2.5 Global filer profile interpretation

The interpretation of electrophoretic data was carried out according to the national Ge.F.I. recommendations [18]. DNA profiles were classified in incomplete profiles: <10 STR loci detected; single source profiles: ≥10 STR loci successfully amplified and characterized by no more than two alleles at each locus; mixed profiles: ≥10 STR loci successfully amplified with more than two alleles detected in at least two different loci.

### 2.6 Statistical analyses

Descriptive statistics were provided, including median and interquartile range (IQ) for the whole sample and for each item. A comparison of the human DNA level recovered pre‐ (V1–V3) and post‐COVID‐19 pandemic cleaning and decontamination plan (V4–V9) was performed by nonparametric paired *t*‐test. The comparison was performed on the whole sample, as well as by separately considering surfaces of the postmortem examination facility and dissection instruments. A multiple nonparametric comparison was also performed for each item.

A *p* < 0.05 was set for significance. Statistical analysis was performed by prism (version 8.2.1., graphpad software, Inc.).

## 3 RESULTS AND DISCUSSION

It is well known that human DNA contamination of evidentiary trace samples has significant detrimental consequences for forensic investigations, deeply affecting the detection of relevant DNAs and complicating the interpretation of genetic results. In this context, our study aimed to evaluate the presence of human background DNA in the local postmortem examination facility, several years after the issue of DNA contamination has been first highlighted, in order to assess whether the increased attention devoted to the decontamination from viruses and pathogens due to the pandemic emergency might have led to a minimization of the DNA contamination risk.

**FIGURE 2:**
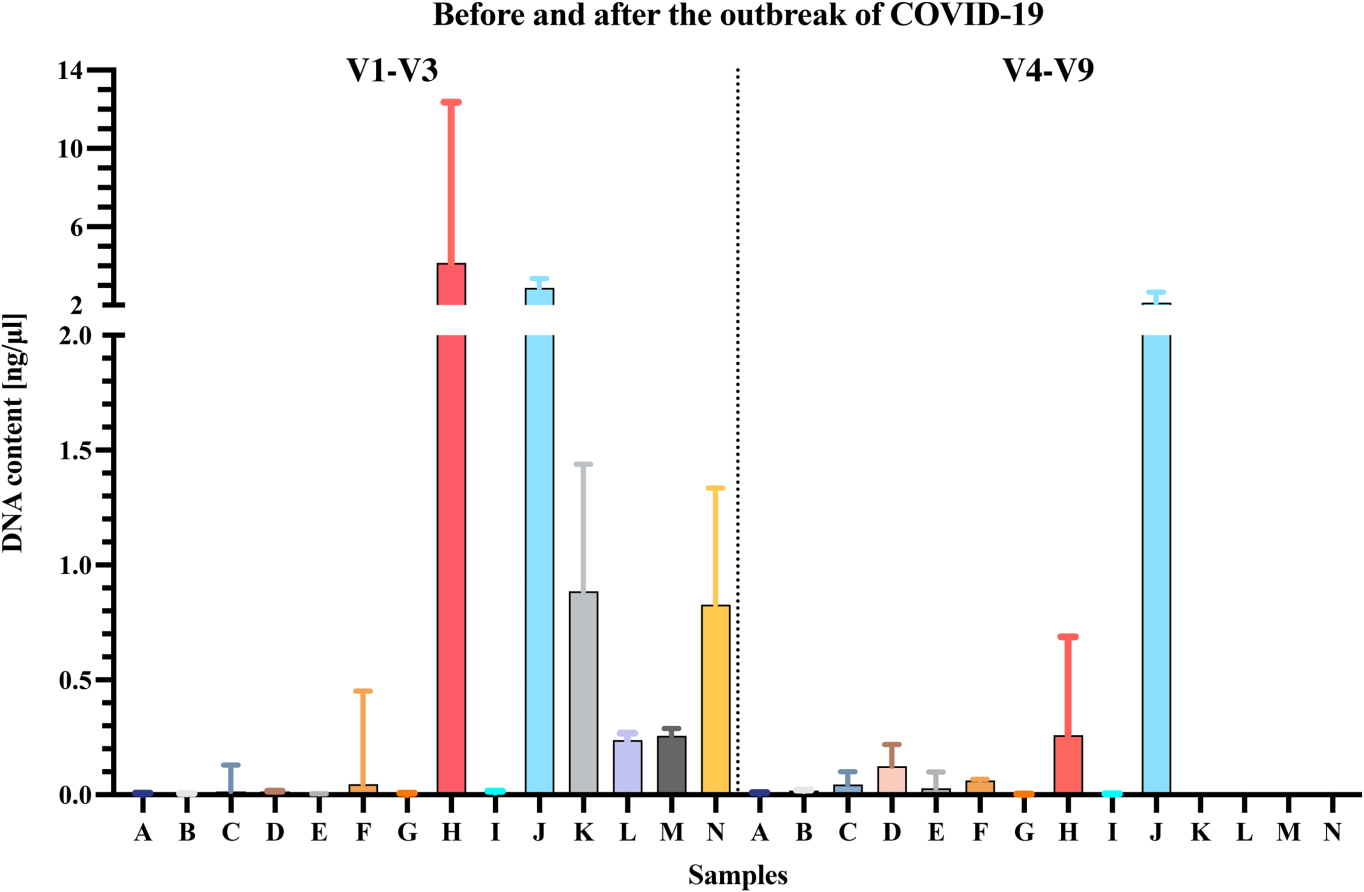
DNA content of samples collected at the local postmortem examination facilities before (V1–V3, on the left side) and after (V4–V9, on the right side) COVID‐19 decontamination plan. For better visualization of lower quantification values, the y axis was divided into two segments: 0–2.0 ng/μl and 2–15 ng/μl. A: autopsy table at the head level; B: autopsy table at the buttocks level; C: autopsy table at the right‐hand level; D: autopsy table at the left‐hand level; E: removable table; F: water tap; G: side table; H: measuring tape; I: glove box; J: head/neck support; K: coronary artery scissor; L: large‐size scissors; M: kocher; N: forceps.

A total number of 91 trace samples were analyzed, of which 38 were collected before the implementation of the COVID‐19 decontamination plan and 53 afterward. Overall, 67 samples (73.6%) resulted in quantifiable human DNA, ranging from 1 pg/μl to 12.4 ng/μl. This rate of positive sample, slightly higher than previously reported [19], might be also due to the higher sensitivity of DNA analysis techniques achieved in the last years [11]. The median human DNA content of the whole sample was 0.007 ng/μl (IQ = 0.136–0.000). Detailed results for surfaces and instruments are shown in Table 2 and Figure 2.

**TABLE 2:** Comparison of human DNA quantity before (V1–V3) and after (V4–V9) the outbreak of the Covid‐19 pandemic

	Item	V1–V3 Median (IQ) [ng/μl]	V4–V9 Median (IQ) [ng/μl]	*p* value
*Surfaces*	*A*	0.001 (0.007–0.001)	0.003 (0.009–0.001)	
	*B*	0.004 (0.003–0.001)	0.017 (0.020–0.011)	
	*C*	0.014 (0.130–0.001)	0.045 (0.100–0.005)	
	*D*	0.013 (0.014–0.003)	0.124 (0.219–0.029)	
	*E*	0.003 (0.004–0.002)	0.028 (0.099–0.014)	
	*F*	0.046 (0.452–0.043)	0.063 (0.068–0.038)	
	*G*	0.001 (0.007–0.001)	0.001 (0.003–0.000)	
	*H*	4.151 (12.370–0.764)	0.260 (0.688–0.142)	
	*I*	0.011 (0.015–0.007)	0.260 (0.688–0.142)	
	*J*	2.877 (3.353–1.014)	2.133 (2.665–0.425)	
*All surfaces*	*A–J*	*0.003 (0.011–0.003)*	*0.048 (0.184–0.004)*	*0.574*
*Dissection instruments*	*K*	0.886 (1.439–0.333)	0.000(0.000–0.000)	
	*L*	0.238 (0.268–0.208)	0.000 (0.000–0.000)	
	*M*	0.257 (0.289–0.225)	0.000 (0.000–0.000)	
	*N*	0.828 (1.335–0.321)	0.000 (0.000–0.000)	
*All dissection instruments*	*K–N*	*0.543 (0.872–0.243)*	*0.000 (0.000–0.000)*	0.029*
*Surfaces and instruments*	*A–N*	*0.114 (0.843–0.004)*	*0.012 (0.073–0.000)*	*0.052*

*Note*: Results are shown as median and interquartile range (IQ). A: autopsy table at the head level; B: autopsy table at the buttocks level; C: autopsy table at the right‐hand level; D: autopsy table at the left‐hand level; E: removable table; F: water tap; G: side table; H: measuring tape; I: glove box; J: head/neck support; K: coronary artery scissor; L: large‐size scissors; M: kocher; N: forceps. V = visit.

* Statistically significant.

All the 67 amplified samples showed 56 (83.6%) mixed profiles of which 44 (78.6%) contained DNA from three or more contributors (data not shown); nine profiles (13.4%) were single source and 2 (3.0%) were incomplete. Among mixed profiles, 48 were generated from samples collected from the surfaces and eight from dissecting instruments. Detailed results are shown in Table 3.

**TABLE 3:** DNA profiling results obtained from surfaces and dissection instruments pre‐ (V1–V3) and post‐COVID‐19 decontamination plan (V4–V9).

	V1–V3			V4–V9		
	Mixed profiles	Single profiles	Both On the number of samples typed	Mixed profiles	Single profiles	Both On the number of samples typed
Surfaces	22	7	29/30 (96.7%)	26	2	28/30 (93.4%)
Dissection instruments	8	0	8/8 (100%)	0	0	0/23 (0%)
All samples	30	7	37/38 (97.4%)	26	2	28/53 (52.8%)

V = visit

### 3.1 Surfaces

Contaminating DNA was found on all the work surfaces of the local facility (*n* = 30/30, 100%), before the implementation of the COVID‐19 cleaning procedures (V1–V3). Lower DNA values were shown for the autopsy table, the side table, and the glove box and higher ones for the water tap, the measuring plastic tape, and the head/neck support. All these surfaces might represent potential sources of contaminating DNA. Indeed, DNA transfer might occur from the autopsy table to the corpse and to the clothes, and later to the evidentiary trace samples. This transfer is particularly dangerous when it involves those areas usually sampled to identify the perpetrator, particularly the fingernails as well as the skin near bruises, bites, and wounds. Therefore, we focused our attention on the surfaces of the table corresponding to the skin of the head, the buttocks, and the hands of the victim. As shown by our results, the autopsy table was not the primary potential source of contaminating DNA, since the median DNA content was in the picogram range. However, the highest level reached was 124 pg/μl for the autopsy table at the left‐hand level, which is certainly enough to obtain a full DNA profile.

Another body area of interest for DNA profiling and for the investigation of criminal activity is represented by the neck, where the skin might be swabbed especially near strangulation marks or scratches. High levels of human background DNA were detected from the head/neck support. This might be due to the poor quality of cleanliness of a surface which is not flat. The issue is particularly relevant in the case of head/neck supports made of hard plastic or wood, which could get nicked or cut by a saw, creating spaces that would protect bits of tissue, that get into that narrow space, from thorough cleaning. Moreover, since the head/neck support is a removable surface, it might not be subjected to the same care as fixed surfaces and escape the cleaning protocols.

In our study, the measuring tape showed an “out of range” DNA level before the outbreak of the pandemic. The surface of the measuring tape is commonly placed in contact with the corpse for the photo‐documentation of injuries and might, thus, act as a vector of contamination during the early phases of the autopsy. For example, if a skin area containing a hematoma is swabbed just after measuring it with the tape and taking of the picture, the DNA profile might contain contaminating DNA from the instrument.

Other surfaces, such as the side table, the water tap surface, and the gloves box showed only low amounts of human DNA, and this result is consistent with the absence of regular contacts with corpses and bare hands. Indeed, gloves should be used not only for hygiene‐related purposes or as personal protection equipment, but also aiming to prevent or reduce the deposit and transfer events of extraneous DNA on objects and surfaces. Nevertheless, contamination might still occur from these surfaces by secondary DNA transfer events [20, 21].

By STR typing, the vast majority of samples with quantifiable human DNA (96.7%) led to the identification of single or mixed profiles, with a predominance of the latter. Given the presence of multiple contributors on the swabbed surfaces and given the high number of autopsied bodies, the mixed profiles are likely explained by multiple DNA transfer events. A further transfer of this contaminating DNA to evidentiary trace samples could make the interpretation of these DNA profiles even more challenging, by leading from a single source to a mixed profile or by increasing the number of contributors.

On the other hand, the presence of single profiles on surfaces also appears worrisome, since this might also appear as the main contributor of a mixed profile obtained from an evidentiary trace sample collected in the autopsy room, masking the DNA of a possible perpetrator.

### 3.2 Dissection instruments

All the samples collected from dissecting instruments showed human quantifiable DNA before the pandemic (V1–V3) (*n* = 8/8, 100%). Dissecting instruments are usually employed at a later stage of the postmortem examination, when all the many evidentiary trace samples have already been collected. However, scissors could be improperly used to collect fingernails samples. Moreover, the dissection instruments could accidentally come in contact with the skin surface of the deceased, which could be swabbed for genetic analysis, or lead to secondary and tertiary DNA transfer. Indeed, they have been shown to act as contamination vectors in the autopsy room, as already reported [11, 19].

In our study, before the COVID‐19 pandemic, each instrument showed a high DNA amount and was thus considered a potential source of contamination (Table 2). Moreover, by STR typing, it was shown that each swab of the dissection instruments led to the identification of mixed profiles, suggesting that multiple DNA transfer events, likely from one postmortem examination to another, took place. This represents a further confirmation of the fact that disposable scissors should be preferred to collect forensic genetic samples in the autopsy room, and that, to avoid secondary or tertiary transfers, the contamination of other instruments should be prevented by cleaning procedures.

### 3.3 Pre‐ and post‐COVID outbreak and cleaning procedures

After the implementation of COVID‐19 cleaning procedures, 29 out of 53 (54.7%) samples taken during V4–V9 resulted in quantifiable human DNA. Particularly, all the samples collected from dissecting instruments (*n* = 23) showed no quantifiable human DNA. Median DNA level across all samples was 0.012 ng/μl (IQ = 0.073–0.000). Detailed results and a graphical representation of DNA quantification values obtained for samples A to N are shown in Tables 2, 3, and Figure 2.

No inhibition or degradation was observed for all analyzed swabs.

By comparing samples collected pre and post‐COVID‐19 decontamination plan, no statistically significant difference in DNA content was found for the whole sample (*p* = 0.052) as well as for the facility surfaces (*p* > 0.05); for dissecting instruments, a statistically significant decrease in DNA content was found (*p* < 0.05).

The cleaning procedures of work surfaces adopted during the pandemic are known to be effective for bacteria, fungi, and even for high hazard viruses [16, 17]. In our study, human quantifiable DNA was still detectable with comparable levels even after the addition of a new cleaning agent and no statistically significant decrease in DNA amount between pre‐ and post‐COVID decontamination plans was seen for surfaces. Hypochlorite‐based detergents, which are effective in DNA removal at a concentration of at least 1% [11, 22, 23], were used in our facility at a concentration of 0.1%, according to the recommendations of the WHO specifically developed for COVID‐19 [12]. The inefficacy in DNA removal was expected, given its dilution and was shown even before the pandemic. With regard to the orthophosphoric acid, the new cleaning agent introduced during the pandemic, on the basis of the results of our study and at the concentrations used (0.2%), this also appeared to be ineffective in the DNA removal. The use of sodium hypochlorite at a higher concentration or of chlorhexidine gluconate might solve both the issues of disinfection from pathogenic germs and of human DNA contamination.

When considering mobile surfaces, such as the measuring tape and the head/neck support, which are constantly handled and put in contact with corpses, the high DNA level only slightly decreased during V4–V9, highlighting that the attention paid to these tools during the cleaning procedures might not be sufficient. Moreover, in our facility, the decision to use a disposable measuring tape has already been put in place.

For dissection instruments, the implementation of the COVID‐19 decontamination plan with chlorhexidine gluconate and autoclave resulted in undetectable DNA content by q‐PCR.

Autoclave has been shown to not be completely effective in DNA removal [10], so that chlorhexidine gluconate likely played a major role in the undetectability of DNA on dissection instruments. According to our results, it might also be useful for the cleaning of those work surfaces that showed unaltered contamination level after the pandemic outbreak despite the COVID‐19‐related cleaning procedures.

### 3.4 Limitations

One limitation is that samples were not taken directly on the body areas usually swabbed for trace DNA evidence collection, but only on surfaces and dissection instruments in the autopsy room. We acknowledge, that, in order to be forensically significant, human contaminant DNA present on these surfaces must be transferred to the body in a quantity sufficient to then be transferred to a swab. However, even small amounts of DNA from transfer events could interfere with data interpretation. In selected cases, it could be useful to preliminarily swab the surfaces on which the body will be placed, as background control.

In addition, as previously discussed, even if the dissection instruments are not used for collection of DNA evidence, which typically is performed before any dissection, they could represent a vector for secondary transfer events and were for this reason included in this study.

The high number of observed mixed profiles indicates that more than one individual contributed to the background DNA of the surfaces and instruments, possibly by primary or higher‐order DNA transfer. By using software for statistical interpretation [24], profiles could be deconvoluted and compared to a database containing reference profiles, as would be done when performing a staff elimination search in forensic genetics laboratory. An elimination database for exclusionary purposes, which has been proposed to identify the source of contamination, was not available for our postmortem facility and this is acknowledged as a major limitation.

Nevertheless, an elimination database would not be sufficiently informative to interpret the complex DNA mixtures which originated from more than three contributors, which, as reported in our study, represented a frequent occurrence, given the cumulative effect of the DNA contamination over time.

The challenges represented by DNA decontamination, the large number of dead bodies and living persons who pass through the autopsy room, and the nature of the autopsy process, all make it unreasonable to expect that the autopsy room could ever be a completely DNA‐free environment, as opposed to clinical or forensic PCR laboratories. The awareness that the autopsy room should be considered a potentially contaminated environment, similar to a crime scene or a transport vehicle, suggests the need to adopt dedicated strategies for trace DNA collection in the autopsy setting to serve as guidelines for best practice policy.

In conclusion, environmental decontamination strategies adopted during the COVID‐19 pandemic only partially solved the long‐standing issue of DNA contamination of postmortem examination facilities. However, the pandemic represents an opportunity to further emphasize the need for standardized evidence‐based protocols, to mitigate the risk of potential DNA contamination in the autopsy room. Recommendations include collecting samples for trace DNA prior to any significant manipulation of the body and while it is still in the body bag, using disposable, sterile instruments, using gloves and changing gloves in‐between one sampling and another.

Considering the high sensitivity of forensic genetic analyses and the awareness of the occurrence and consequences of DNA transfer events, the development of an effective protocol requires the involvement of a cross‐disciplinary forensic science team.
